# Quinoa and Peanut Extracts as Alternatives to Animal‐Derived Extracellular Matrix Proteins in Cultured Meat Production

**DOI:** 10.1111/1750-3841.71509

**Published:** 2026-09-27

**Authors:** Dominik Mundt, Kuo‐Hui Chiu, Stefan Schillberg, Che Julius Ngwa

**Affiliations:** ^1^ Fraunhofer Institute for Molecular Biology and Applied Ecology IME Aachen Germany; ^2^ Institute for Molecular Biotechnology RWTH Aachen University Aachen Germany

## Abstract

Cultured meat, produced through the cultivation of animal cells, represents a sustainable and ethical alternative to traditional livestock farming. The cultivation and expansion of animal cells for cultured meat production rely heavily on extracellular matrix (ECM) proteins, which are needed for cell adhesion, proliferation, and differentiation. However, common ECM components such as gelatin, collagen, vitronectin, and Matrigel are mainly derived from animal sources, raising ethical concerns and sustainability issues. In this study, we used in silico analysis to identify edible plant‐derived proteins containing Arg–Gly–Asp (RGD) motifs, which can promote cell adhesion and proliferation like ECM proteins. Peanut (*Arachis hypogaea*) and quinoa (*Chenopodium quinoa*) were identified as promising candidates. Water‐soluble extracts were prepared from the plant seeds and evaluated for their ability to support the adhesion, proliferation, and differentiation of murine C2C12 and immortalized bovine satellite cells. We found that the extracts predominantly contained low‐molecular‐weight proteins, and cell culture surfaces coated with either extract supported cell attachment and proliferation at levels comparable to those achieved with animal‐derived ECM proteins. Analysis of the most abundant proteins in each plant extract as determined by mass spectrometry confirmed the presence of RGD motif‐containing proteins. Subsequently, one RGD‐containing protein identified from each extract was produced using a cell‐free expression system, and lysates containing the produced proteins were evaluated for their ability to support cell attachment and proliferation. The results indicated enhanced cell attachment and proliferation, indicating a potential role of the proteins. Our findings demonstrate that peanut and quinoa protein extracts are promising and inexpensive animal‐free alternatives for surface functionalization in cultured meat applications.

## 1 Introduction

The increasing global population and growing demand for meat products raise significant concerns regarding the environmental and ethical issues associated with livestock farming. Conventional livestock farming significantly contributes to greenhouse gas emissions, land and water use, animal welfare issues, and biodiversity loss (Poore and Nemecek 2018; Steffen et al. 2015; Zhao et al. 2014). Cultured meat, which is produced from cultured animal cells, has therefore emerged as a promising alternative with the potential to reduce the environmental footprint of conventional meat production (Kirsch et al. 2023; Ngwa et al. 2025).

Cultured meat production involves the isolation, expansion, and differentiation of animal stem cells, including muscle and fat precursor cells, using appropriate cell culture media and proteins that support cell attachment. The ideal in vitro environment for these cells is created by using extracellular matrix (ECM) proteins such as gelatin, collagen, fibronectin, vitronectin, and Matrigel, which are mainly derived from animal sources. These proteins feature cell‐binding ligands, most notably the Arg–Gly–Asp (RGD) motif, which interacts with cell‐surface integrins and activates intracellular signaling pathways that regulate cell survival, proliferation, and differentiation (Nieberler et al. 2017). ECM proteins can be used to coat the surfaces of culture dishes or microcarriers for cell expansion in bioreactors or for the development of 3‐dimensional (3D) scaffolds for structured meat production.

Although ECM proteins are effective and commercial food‐grade coatings are available, they are predominantly animal‐derived and therefore do not align with the objective of developing fully animal‐free production pipelines. Plant‐derived proteins have therefore been evaluated as sustainable, scalable, and animal‐free alternatives for surface coatings and scaffold materials (Jing et al. 2021). Certain plant proteins can influence the behavior of muscle cells, including the promotion of proliferation and differentiation. For example, dietary soy proteins influence C2C12 myoblast differentiation (Geurs et al. 2025), and pea protein‐based scaffolds encourage the growth of bovine muscle cells (David et al. 2024). These findings suggest that plant proteins provide bioactive cues relevant to muscle cell–material interactions.

Building on this concept, we screened edible plants for proteins containing RGD motifs, which could be used as surface coatings or scaffolds for cultured meat production. However, other peptide sequences, such as Leu‐Asp‐Val (LDV), which is a ligand for α4β1 integrins, or Leu‐Arg‐Glu (LRE), which promotes the binding of neuronal cells, can also serve as potential surface coatings (Nomizu et al. 1998; Vogelezang et al. 2001). We focused on protein extracts from the seeds of peanut and quinoa and tested their ability to support the adhesion and proliferation of C2C12 myoblasts and bovine satellite cells and their compatibility with myogenic differentiation under standard in vitro conditions. By validating plant protein sources as pro‐adhesive, food‐grade coatings, this study contributes to the development of sustainable, animal‐free scaffold materials for cultured meat production.

## 2 Materials and Methods

### 2.1 In Silico Identification of Edible Plant Proteins With RGD Motifs

Plant proteins were identified by searching the National Center for Biotechnology Information (NCBI) nr database using BLASTP (Basic Local Alignment Search Tool, Protein), focusing on edible plant proteins containing two consecutive RGD sequences (RGDRGD). It is not possible to identify single RGD sequences using the blast tool due to their very short length.

### 2.2 Antibodies and Primers

Primary rabbit antibodies used in this study were obtained from ABClonal (Germany) and were used to detect myoblast determination protein 1 (MyoD) (A0671), myogenin (MyoG) (A6664), and the myosin heavy chain (MyoHC) (A4963). For indirect immunofluorescence assays (IFAs), all antibodies were diluted 1:500. We used as a secondary antibody a goat anti‐rabbit antibody conjugated to Alexa Fluor 594 (Thermo Fisher Scientific, USA; A‐11012). For western blot analysis we diluted the antibodies 1:1000. As a secondary antibody for western blot, we used a goat anti‐rabbit antibody conjugated with alkaline phosphatase (Sigma‐Aldrich, USA; A3812). The primers used in this study are listed in (Table S1).

### 2.3 Preparation of Protein Extracts

Protein extracts were prepared from peanut and quinoa seeds obtained from local stores in Germany. The seeds were ground in liquid nitrogen using a mortar and pestle, and 5 *g* of the resulting powder was resuspended in 100 mL of phosphate‐buffered saline (PBS) (PAN BIOTECH, Germany; P04‐36500). The suspension was incubated at 40°C for 45 min, followed by centrifugation (4415 × *g*, 10 min, room temperature (RT)), and the supernatant was passed through a 0.22‐µm filter to remove contaminants. The protein concentration in the extract was determined using a bicinchoninic acid (BCA) assay kit (Thermo Fisher Scientific; 23227) and adjusted to 1000 µg/mL. Aliquots were subsequently prepared and stored at –20°C.

#### 2.3.1 Surface Coating With Extracts

Petri dishes that do not support cell attachment (SARSTEDT, Germany; 82.1473.001) were coated evenly with 3.15 µg/cm^2^ of aqueous plant protein extract and allowed to dry at 45°C for 48 h. Cell attachment on extract‐coated plates was compared to albumin‐coated plates as well as to non‐coated plates as a negative control, and to plates coated with gelatin or vitronectin as positive controls.

#### 2.3.2 RGD Inhibition Assay

To determine whether proteins carrying RGD sequences were involved in cell attachment, we performed an RGD inhibition assay on peanut‐extract‐coated plates. To this end, 96‐well plates that had not been treated with tissue culture were pre‐coated with either 1% gelatin (type A, bloom 60 g; Fluka, catalogue no. 48720), which served as a positive control, or 0.5% peanut protein extract. The plates were then left to dry overnight at 45°C. C2C12 cells were pre‐treated with RGD peptides (Hycultec, catalogue no. HY‐P0278‐5) or albumin at concentrations of 0, 5, 10, 20, 50, or 100 µg/mL for 1 h at 37°C. Following treatment, the cells were washed with PBS and collected by centrifugation to remove unbound peptides or proteins. The cell pellets were then resuspended in fresh culture medium and seeded onto the pre‐coated plates. Cells were incubated for 3 h at 37°C to allow adhesion. After incubation, the wells were gently washed with PBS to remove non‐adherent cells. The number of adherent cells was subsequently quantified using the Cell Counting Kit‐8 (CCK‐8) assay according to the manufacturer's instructions (Abcam, UK; ab228554).

### 2.4 Cell‐Free Protein Expression

For cell‐free expression of the RGD‐containing proteins, the bifunctional inhibitor protein from peanut and the Cupin type 1 protein from quinoa were selected. For each protein, either a gene fragment encompassing the RGD motifs (Cupin type 1, 176 amino acids) or the entire coding sequence (bifunctional inhibitor protein, 185 amino acids) was synthesized and cloned into the pLenEx vector, which enables cytosolic expression in a cell‐free lysate derived from tobacco BY‐2 suspension cells (Buntru et al. 2022). The resulting plasmid constructs were verified by colony PCR and sequencing prior to their use for cell‐free protein expression. For each construct, the optimal plasmid volume V (µL) was calculated using Formula 1 (see Formula S1). Already prepared and stored BY‐2 lysate (BYL) stocks (Buntru et al. 2015; Buntru et al. 2022) were thawed at room temperature, inverted three times, and then placed on ice. Next, the construct‐specific plasmid volume was added to each well, and the reaction was filled to a total volume of 50 µL with BYL. The reactions were incubated for 48 hours at 25°C, 80% humidity, and 500 rpm on an orbital shaker. Expressed proteins were analyzed by sodium dodecyl sulfate‐polyacrylamide gel electrophoresis (SDS‐PAGE) and western blot under the previously described conditions (Hagiwara 2022).

### 2.5 Cell Cultivation

C2C12 mouse myoblasts (Cytion, Germany; 400476) were maintained in Dulbecco's modified Eagle's medium (DMEM) (PAN BIOTECH; P04‐03550) supplemented with 10% (v/v) fetal bovine serum (FBS) (PAN BIOTECH; P30‐3031) and 1% (v/v) penicillin/streptomycin (PAN BIOTECH; P06‐07100) (DMEM+) at 37°C, in a 5% CO_2_ atmosphere. The cells were passaged at 70% confluence by removing the spent culture medium, washing with 10 mL PBS, and incubating with 5 mL trypsin (PAN BIOTECH; P10‐023100) at 37°C for 10 min for cell detachment. We then added 5 mL of DMEM+ and transferred 1:40 of the cell suspension to a new T75 culture flask, which was replenished with 20 mL of fresh DMEM+.

The immortalized bovine satellite cell line (iBSCs, kerafast, USA; ETU009) was maintained in DMEM supplemented with 20% (v/v) FBS, 1 ng/mL FGF2 (Thermo Fisher Scientific; NJ 08512), and 1% (v/v) penicillin/streptomycin (DMEM+F) as above. The cells were passaged every second day as above, but 5 mL of Accutase (PAN Biotech) rather than trypsin was used for cell detachment, and 1500 cells/cm^2^ were seeded into each new T75 culture flask. Before seeding, the flask was coated with vitronectin (Gibco; A400457) for 1 h.

#### 2.5.1 Cell Viability/Proliferation Assay

We seeded 5 × 10^4^ cells in 5 mL medium into Petri dishes coated with each plant protein extract and incubated at 37°C in a 5% CO_2_ atmosphere for 5 days with a medium change on day 3. Cell attachment and proliferation were monitored using the cell counting kit‐8 (CCK‐8) assay (Abcam, UK; ab228554). Briefly, this assay measures cell viability by quantifying the conversion of WST‐8 (2‐(2‐methoxy‐4‐nitrophenyl)‐3‐(4‐nitrophenyl)‐5‐(2,4‐disulfophenyl)‐2H‐tetrazolium monosodium salt) into a colored formazan product by endogenous dehydrogenases. The intensity of the orange, water‐soluble formazan dye is proportional to the number of viable cells and was measured by spectrophotometry at 450 nm in a CLARIOstar microplate reader (BMG LABTECH, Germany). The plates coated with plant proteins were compared to the uncoated negative control and the gelatin or vitronectin positive controls for C2C12 and iBSCs, respectively. Bright‐field images were analyzed using ImageJ (Schneider et al. 2012).

#### 2.5.2 Induction of Cell Differentiation

We seeded 5 × 10^4^ cells on petri dishes coated with plant protein extracts as above and cultivated them for 2 days under standard conditions. The medium was then replaced with differentiation medium (DMEM supplemented with 2% (v/v) horse serum (PAN BIOTECH; P30‐0702) for C2C12 myoblasts and 2% (v/v) FBS for iBSCs) to induce myogenesis (Stout et al. 2023). The cells were cultured in differentiation medium for 8 or 12 days, with medium replacement every 3 days. Myogenic differentiation was periodically evaluated by microscopy before the cells were harvested.

### 2.6 RNA Extraction and Analysis

Cultured cells were washed with PBS and detached as described above. The cell pellets were suspended directly in 20 volumes of pre‐warmed Trizol reagent (Invitrogen, USA) and incubated at 37°C for 5 min, shaking at 300 rpm. Total RNA was isolated as previously described (Hanhsen et al. 2022). RNA quality was assessed using a NanoDrop c spectrophotometer. We prepared cDNA from 1 µg of total RNA using the First‐Strand cDNA Synthesis Kit (Thermo Fisher Scientific; 18091050). The cDNA was initially assessed for genomic DNA contamination by PCR using primers specific for the *GAPDH* or *β‐actin* genes (Table S1). Control reactions lacking reverse transcriptase were included to exclude genomic DNA contamination.

Diagnostic RT‐PCR and quantitative reverse transcription polymerase chain reaction (RT‐qPCR) were carried out using primers listed in Table S1. RT‐qPCR was carried out in triplicate using the q‐TOWER system (Analytik Jena, Germany). Each 10‐µL reaction contained Maxima SYBR Green qPCR Master Mix (A&A Biotechnology). The relative transcript expression levels were subsequently calculated using the 2^−ΔCt^ method (Livak and Schmittgen 2001), with the endogenous control gene [GAPDH (C2C12 myoblast) and β‐actin (iBSC)].

#### 2.6.1 Immunofluorescence Assay

Following 2D differentiation, cells were fixed with 4% (w/v) paraformaldehyde for 15 min at RT, then washed three times with PBS and quenched with 100 mM glycine for 10 min. Cells were permeabilized with 1% (v/v) Triton X‐100 for 30 min at RT. Nonspecific binding sites were blocked with 0.5% (w/v) albumin fraction V (Capricorn Scientific, Germany; BSA‐1S) and 1% (w/v) skimmed milk for 1 h at RT. The cells were incubated with primary antibodies (See Antibodies and Primer) overnight at 4°C, then washed three times with PBS containing 0.1% (v/v) Tween‐20 (PBS‐T). Alexa Fluor 594‐conjugated secondary antibodies (Thermo Fisher Scientific; A‐11012) were applied for 1 h at RT. Filamentous actin (F‐actin) was stained with 2 µM phalloidin‐FITC (Sigma‐Aldrich; P5282) for 1 h at RT. Nuclei were counterstained with 4′,6‐diamidino‐2‐phenylindole (DAPI) (Carl Roth, Germany; 6335.1) for 20 min at RT. Finally, the cells were washed five times with PBS‐T and mounted. Images were acquired using a BZ‐X800 Analyzer (Keyence, Japan).

#### 2.6.2 Western Blot

Lysates were prepared from differentiated cells on coated dishes. Cells were washed twice with cold PBS and resuspended in cold RIPA buffer (50 mM Tris‐HCl pH 8.0, 150 mM NaCl, 1% (v/v) Triton X‐100, 0.5% (w/v) sodium deoxycholate, 0.1% (w/v) SDS, then lysed using three 30‐second cycles in an ultrasonic bath at 4°C. Samples were mixed with 5 × SDS loading buffer at a 5:1 ratio, heated for 5 min at 95°C, and separated by SDS‐PAGE. The separated proteins were transferred to a nitrocellulose membrane (Carl Roth) according to the manufacturer's protocol. Membranes were rinsed in 10 mM Tris‐HCl pH 7.5 containing 0.1% (v/v) Tween‐20 (TBS‐T) and blocked in TBS containing 5% (w/v) skimmed milk and 1% (w/v) albumin for 1 h at room temperature. Membranes were incubated overnight at 4°C with primary antibodies (see Antibodies and primers), washed, and incubated with alkaline phosphatase‐conjugated anti‐rabbit or anti‐mouse IgG secondary antibodies (Sigma‐Aldrich) for 1 h at RT. Bands were developed with NBT/BCIP (Thermo Fisher Scientific; 34042). Blots were scanned and analyzed using ImageJ.

### 2.7 Mass Spectrometry and Proteomic Analysis of Peanut and Quinoa Extracts

Peanut and quinoa seed protein extracts were prepared in PBS as described above. Protein concentrations were determined using a bicinchoninic acid (BCA) assay (Thermo Scientific). For each sample, a final concentration of 1 M urea was achieved by adjusting 100 µg of protein lysate in ammonium bicarbonate (ABC). Proteins were reduced with 4 mM dithiothreitol (DTT) in 50 mM ABC for 30 min at 56°C, followed by alkylation with 10 mM iodoacetamide (IAA) in 50 mM ABC for 30 min at room temperature in the dark. Proteins were subsequently digested with MS‐grade trypsin (Trypsin Protease, MS Grade; cat. no. 90058; Thermo Scientific) at an enzyme‐to‐protein ratio of 1:100 (w/w) for 16 h at 37°C. The resulting peptides were desalted using in‐house‐prepared C18 tips and dried in a vacuum concentrator. Prior to LC–MS/MS analysis, peptides were resuspended in 3% formic acid (FA) and 1% acetonitrile (ACN).

LC‐MS/MS analysis was carried out by first trapping the peptides on a trapping column (Acclaim PepMap100, C18, 5 µm, 100 Å, 300 µm i.d. × 5 mm, Thermo Scientific) on a Vanquish Neo LC system (Thermo Scientific). The peptides were then separated on an analytical column at 45°C (Aurora Ultimate 25*75 C18 UHPLC column, IonOpticks, Australia) with the following gradient: 0–1 min: 0%–2% buffer B (buffer A: 0.1% FA; buffer B: 80% ACN, 0.1% FA), 1–12 min: 2–3% buffer B, 12–120 min: 2–25% buffer B, 120–143 min: 25–40% buffer B, 143–148 min: 40%–95 % buffer B, 148–153 min: 99% buffer B, 153–155 min: 95%–2% buffer B, 155–160 min: 2% buffer B.

Data were acquired on the Exploris 480 mass spectrometer (Thermo Scientific) by using a data‐independent acquisition (DIA) method (Lambertz et al. 2025) with staggered acquisition windows (isolation window of 8 m/z, shift of 4 m/z). MS1 scans: 390–1010 or 394–1014 m/z. MS settings: resolution 60k, normalized AGC target: 100%, max. injection time mode: 1; DIA settings: resolution 30k, normalized HCD collision energy: 30%, normalized AGC target: 1000%, max. injection time: 55 ms.

Raw data were processed using Spectronaut 19 with stringent identification settings as described before (Lambertz et al. 2025). The intensity‐based absolute quantification (iBAQ) values were used as the main output of the raw data analysis. The UniProt databases used were peanut: UP000289738 for *Arachis hypogaea* and quinoa: UP000596660 for *Chenopodium quinoa*. Both databases were extended with a universal contaminants database (Frankenfield et al. 2022).

The resulting protein files were imported into the Perseus version 2.1.3.0 (Tyanova and Cox 2018) with the iBAQ values as the primary columns. The lists were filtered for a minimum of 2 unique peptides (Spectronaut entry PG.NrOfStrippedSequencesIdentified), and a protein was required to be present in all three technical replicates (based on iBAQ values).

To functionally annotate and analyze the proteins identified in the peanut and quinoa extracts using mass spectrometry, the protein identifiers (hits) derived from the raw data were entered into the UniProt Retrieve/ID Mapping Tool (https://www.uniprot.org/id‐mapping). The identified proteins were annotated using Gene Ontology (GO) terms corresponding to the three main GO categories: Biological Process, Molecular Function, and Cellular Component. The resulting annotation data were downloaded from UniProt in Excel format and subsequently analyzed to characterize the functional profiles of the identified proteins. In addition, the 15 most abundant proteins from each extract were selected for further analysis and examined for the presence of motifs associated with cell adhesion.

### 2.8 Statistical Analysis

Experiments were performed in three independent replicates (biological replicate (N_b_)), each with three technical replicates (N_t_). Data were analyzed using GraphPad Prism v10.0. Significance differences as indicated by asterisks on the figure were *p ≤ 0.05, **p ≤ 0.01, ***p ≤ 0.001, and ****p ≤ 0.0001.

## 3 Results

### 3.1 In Silico Analysis for Protein Identification

An in silico search was conducted using BLASTP against the NCBI Non‐Redundant (nr) database to identify proteins from edible plants containing a tandem RGD motif (RGDRGD). Five species were identified: peanut (*Arachis hypogaea*), quinoa (*Chenopodium quinoa*), potato (*Solanum tuberosum*), rapeseed (*Brassica napus*), and wheat (*Triticum aestivum*) (Table 1). The species were analyzed for total proteins and RGDRGD motif‐containing proteins. The number of identified RGDRGD motif‐containing proteins among the species ranged from 5 to 74. Given the broad availability of peanut and quinoa seeds and their multiple RGDRGD‐containing proteins, these species were selected for the preparation of aqueous protein extracts for downstream analyses.

**TABLE 1:** In silico analysis of 5 commonly used edible plants with ‐RGDRGD‐containing proteins.

Plant	Total number of proteins	‐RGDRGD‐ containing proteins
Peanut (*Arachis hypogaea*)	744,616	70
Quinoa (*Chenopodium quinoa*)	64,877	20
Potato (*Solanum tuberosum*)	205,559	72
Rapeseed (*Brassia napus*)	633,662	74
Wheat (*Triticum aestivum*)	484,850	5

Further analysis of the peanut proteome identified 744,616 genes, 59.7% of which were classified as protein‐coding genes. These included 70 amino acid sequences containing one or more RGD motifs, indicating a wide availability of potentially bioactive proteins. Although these findings suggest that peanut‐derived proteins may be suitable for surface functionalization, their presence and accessibility in protein extracts would need to be validated. For quinoa, 64,877 genes were identified, representing 83.6% of the annotated protein‐coding genes. Among these, 20 proteins contain RGDRGD motifs. Together, the in silico results supported the selection of peanut and quinoa for further experimental investigation.

To assess the relationship between the computationally identified proteins and the experimentally accessible protein fractions, aqueous protein extracts from peanut and quinoa were subsequently characterized. Samples collected at different stages of the extraction process were analyzed with respect to protein yield and molecular weight distribution. Gelatin was included as a positive control due to its RGD‐containing sequence, and albumin, which does not have an RGD motif, was used as a negative control.

**FIGURE 1:**
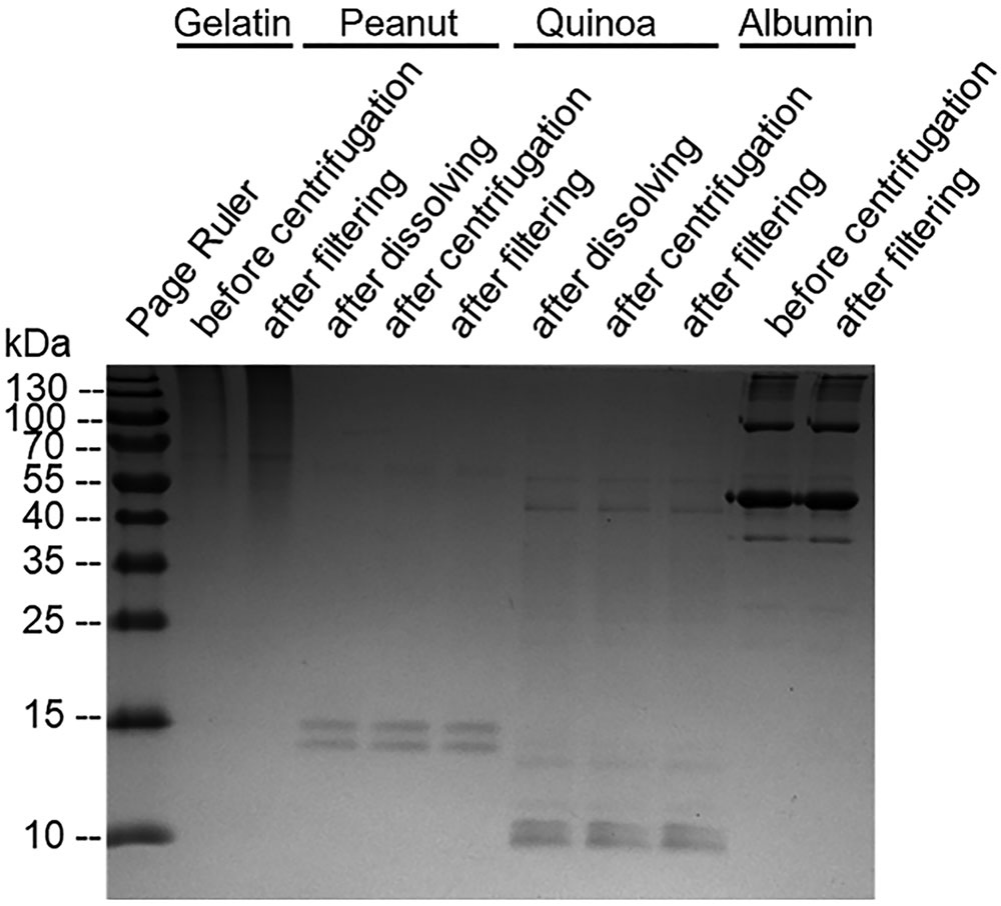
SDS‐PAGE analysis of protein extracts during different extraction steps. Line 1: Page Ruler protein ladder, Line 2: Gelatin before centrifugation, Line 3: Gelatin after sterile filtration, Line 4: Peanut protein extract before centrifugation (after dissolving in water), Line 5: Peanut protein extract after centrifugation, Line 6: Peanut protein extract after sterile filtration, Line 7: Quinoa protein extract before centrifugation, Line 8: Quinoa protein extract after centrifugation, Line 9: Quinoa protein extract after sterile filtration, Line 10: Albumin before centrifugation, Line 11: Albumin after sterile filtration.

SDS‐PAGE analysis revealed distinct protein profiles for the control and the plant‐derived protein extracts (Figure 1). Gelatin and albumin controls displayed protein bands spanning a molecular weight range of ∼35–140 kDa, which remained detectable following centrifugation and filtration. Peanut extracts yielded protein bands consistently between 10 and 25 kDa, with the most prominent band at ∼15 kDa. Additional faint bands were detected at ∼55 kDa. There was negligible variation in band intensity across sampling time points during the extraction process. Similarly, quinoa extracts yielded protein bands mostly in the size range of 10–15 kDa and 40–50 kDa in all samples. Additional faint bands were detectable in the ∼15–40 kDa and 25–70 kDa ranges, suggesting the presence of less abundant protein fractions with intermediate and higher molecular weights. Overall, these bands were less pronounced than those observed in the peanut extracts (Figure 1).

### 3.2 Plant‐Based Protein Extract Coatings Enhance Cell Adhesion in C2C12 Myoblasts and iBSCs

To initially assess whether the protein extracts support cell attachment and proliferation, we initially tested the purified extracts for cell attachment and proliferation without determining the protein concentration. Non‐cell binding Petri dishes were coated with 5 mL of 1% (w/v) of peanut and quinoa extract solutions, which were then left dry. C2C12 cells were then seeded and cultured for 5 days. Microscopy analysis indicated high cell attachment and proliferation on both the peanut‐ and quinoa‐coated dishes as compared to non‐coated and albumin‐coated controls (Figure S1A).

Next, we sought to identify protein concentrations that optimally supported cell attachment and proliferation. Coating concentrations ranging from 0 to 7.86 µg/cm^2^ were evaluated using the CCK‐8 metabolic assay, with values normalized to the uncoated control. For gelatin‐coated Petri dishes, no significant differences were observed among the tested coating concentrations. However, we observed a ∼40% increase in cell number in the coated samples compared to uncoated surfaces (Figure S1B).

Similarly, the peanut protein coatings resulted in higher cell numbers across the tested concentration range of 0.15 to 7.86 µg/cm^2^. Although no statistically significant differences were detected among the individual coating concentrations, cell numbers increased by approximately 50% compared to the uncoated control (Figure S1B).

In contrast, quinoa protein coatings showed a different concentration‐dependent response. At the highest coating concentration (7.86 µg/cm^2^), ∼25% fewer viable cells were detected compared to the uncoated control. No statistically significant differences were observed among concentrations ranging from 0.15 to 3.14 µg/cm^2^. These findings suggest that in contrast to peanut protein coatings, which supported increased cell numbers across a broad concentration range, quinoa protein coating may have a narrow effective range for supporting cell attachment and proliferation or may contain a high concentration of impurities such as saponins, which inhibit cell proliferation.

**FIGURE 2:**
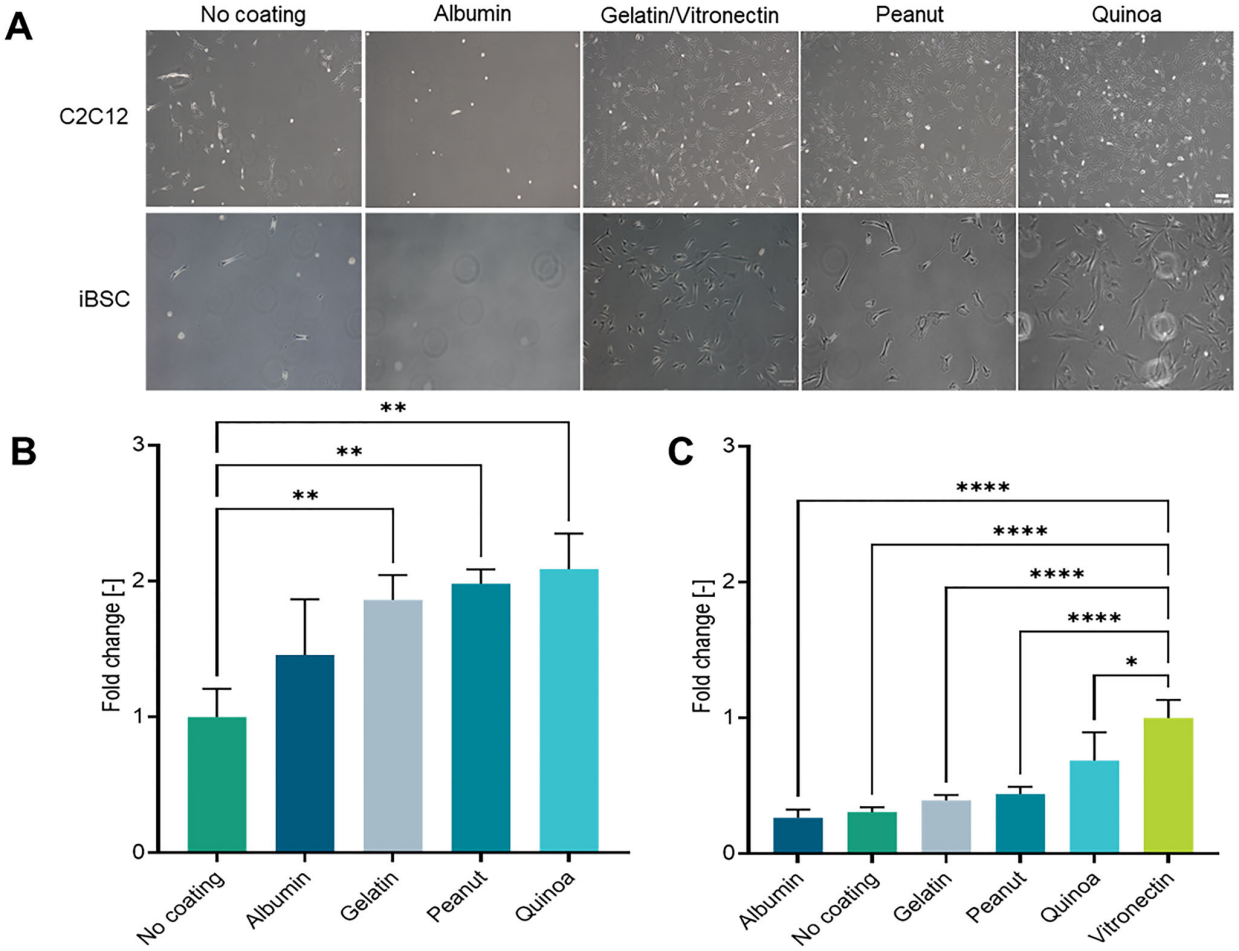
C2C12 and iBSC attachment and proliferation on peanut and quinoa extract‐coated surfaces. A: Representative microscopic image of C2C12 myoblasts and iBSC after proliferation assay on plant protein‐coated Petri dishes over five days. Cell proliferation was measured using the CCK‐8 assay after the proliferation of C2C12 myoblasts (B) and iBSC (C) on plant protein‐coated Petri dishes after five days. Results are normalized to “no coating” or vitronectin and are given as ± standard deviation. *N*_b_ = 3. *p < 0.05, **p < 0.01, ****p < 0.0001. Scale bar: 100 µm.

Having identified 3.14 µg/cm^2^ as a suitable coating concentration, this concentration was used in further experiments to evaluate the ability of the plant coatings to support cell attachment and proliferation over an extended culture period. After 5 days of culture, C2C12 myoblasts grown on albumin‐coated plates exhibited reduced viability and adhesion, as evidenced by confluence levels of ∼30% with cells exhibiting a predominantly rounded morphology. Similarly, only minimal cell attachment was observed on uncoated plates, indicating that neither condition adequately supported sustained C2C12 cell attachment and proliferation. In contrast, gelatin‐coated plates achieved ∼70% confluence with predominantly physiological morphology, the peanut protein coating achieved ∼80% confluence with predominantly normal morphology, and the quinoa protein coating achieved ∼95% confluence with morphology comparable to gelatin (Figure 2A).

To determine whether these effects extended to a cell type relevant to cultured meat production, a similar experiment was performed using iBSCs (Figure 2A). After 5 days of culture, both albumin‐coated and uncoated plates exhibited low cell attachment, reaching only ∼10% confluence with predominantly rounded cells and few adherent cells. A high proportion of adherent iBSCs was observed on gelatin (∼20% confluence, star‐shaped morphology); however, a considerable proportion of non‐adherent cells were observed in suspension. Peanut protein‐coated surfaces resulted in slightly higher (∼25% confluence) than gelatin‐coated surfaces (∼50% confluence), with most cells remaining adherent to the surface. Notably, quinoa protein‐coated surfaces supported substantially greater cell attachment and growth, reaching ∼70% confluence, with adherent cells displaying physiological morphology. This was comparable to the confluence achieved with vitronectin (∼70%), which was included as a positive control.

Cell attachment and proliferation on the coated surfaces were further quantified using the CCK‐8 assay, and the values were normalized to the uncoated control group (Figure 2B,C). No statistically significant difference was observed between the albumin‐coated and uncoated groups. In contrast, gelatin, peanut, and quinoa protein coatings increased the cell number by ∼2.5‐fold relative to the uncoated control, with quinoa coating yielding the highest mean value.

For iBSCs, CCK‐8 assay values were normalized with the vitronectin positive control. Quinoa protein‐coated surfaces supported approximately 75% of cell number compared to vitronectin, whereas peanut‐coated surfaces reached approximately 50% of the vitronectin control (Figure 2C).

Our comprehensive evaluation demonstrates that the plant seed protein coatings, particularly those derived from quinoa, can substantially enhance the attachment and proliferation of both C2C12 myoblasts and iBSCs. The ability of the protein coatings to support two distinct myogenic cell types highlights their potential as a plant‐derived alternative to conventional cell adhesives and provides a basis for investigating their effect on subsequent cell differentiation.

### 3.3 Plant‐Based Protein Extract Coatings Enable the Differentiation of C2C12 Myoblasts and iBSCs

**FIGURE 3:**
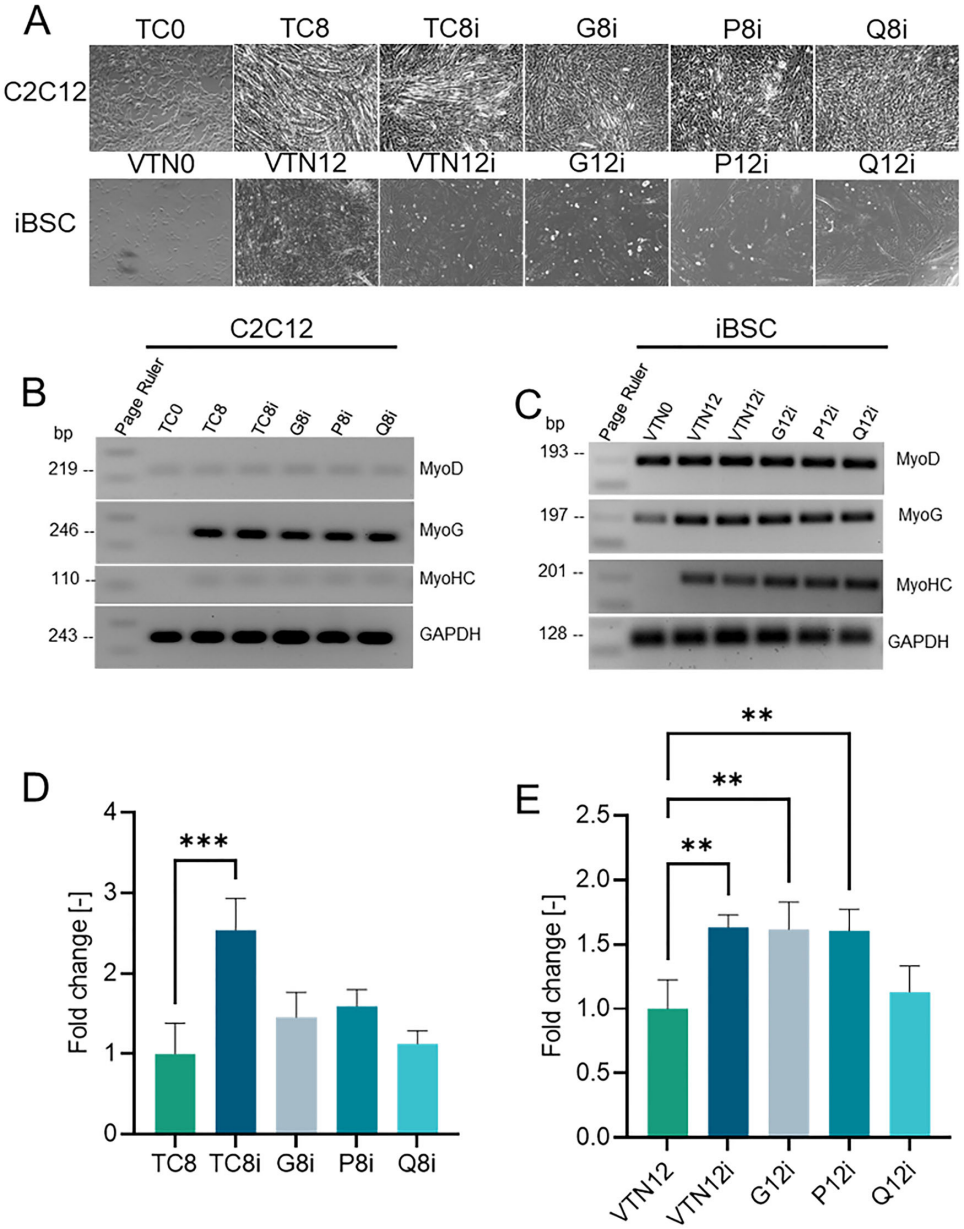
Differentiation of C2C12 and iBSC cells on peanut‐ and quinoa‐extract‐coated surfaces assessed by microscopy, RT‐PCR, and qPCR analysis of myogenic marker expression. A: Microscopic representative image of C2C12 myoblasts after differentiation assay on plant protein, gelatin/vitronectin‐coated Petri dishes over 8 days, and iBSC after 12 days. TC0: Tissue culture plate day 0, TC8: Tissue culture plate day 8 not induced; TC8i: Tissue culture plate day 8 induced; G8i: Gelatin‐coated plate day 8 induced; P8i: Peanut‐coated plate day 8 induced; Q8i: Quinoa‐coated plate day 8 induced. VTN0: Vitronectin‐coated plate day 0, VTN12: Vitronectin‐coated plate day 12 not induced, VTN12i: Vitronectin‐coated plate day 12 induced, G12i: Gelatin‐coated plate day 12 induced, P12i: Peanut‐coated plate day 12 induced, Q12i: Quinoa‐coated plate day 12 induced. Scale bar: 100 µm. (B): Diagnostic RT‐PCR for the detection of muscle differentiation marker MyoD, MyoG, and MyoHC, as well as the housekeeping gene GAPDH in C2C12 myoblasts. TC0: Tissue culture plate day 0; TC8: Tissue culture plate day 8 not induced; TC8i: Tissue culture plate day 8 induced, G8i: Gelatin‐coated plate day 8 induced; P8i: Peanut‐coated plate day 8 induced; Q8i: Quinoa‐coated plate day 8 induced. (C) Diagnostic RT‐PCR for the detection of muscle differentiation marker MyoD, MyoG, and MyoHC, as well as the housekeeping gene GAPDH in iBSCs. VTN0: Vitronectin‐coated plate day 0; VTN12: Vitronectin‐coated plate day 12 not induced, VTN12i: Vitronectin‐coated plate day 12 induced, G12i: Gelatin‐coated plate day 12 induced, P12i: Peanut‐coated plate day 12 induced, Q12i: Quinoa‐coated plate day 12 induced. (D) Quantitative RT‐qPCR analysis of MyoHC expression of C2C12 myoblasts after differentiation assay. TC8: Tissue culture plate day 8 not induced; TC8i: Tissue culture plate day 8 induced; G8i: Gelatin‐coated plate day 8 induced; P8i: Peanut‐coated plate day 8 induced; Q8i: Quinoa‐coated plate day 8 induced. *N*_b_ = 3, ***p < 0.001. (E) Quantitative RT‐qPCR analysis of MyoHC expression of iBSC after differentiation assay. iBSC differentiation over 12 days. VTN12: Vitronectin‐coated plate day 12 not induced; VTN12i: Vitronectin‐coated plate day 12 induced; G12i: Gelatin‐coated plate day 12 induced; P12i: Peanut‐coated plate day 12 induced; Q12i: Quinoa‐coated plate day 12 induced. Results are normalized to TC8 or VTN12 for C2C12 cells and iBSCs, respectively, and are shown as ± standard deviation. *N*_b_ = 3, **p < 0.01.

The differentiation potential of C2C12 myoblasts was examined over an 8‐day period on the different coated surfaces. At the time of induction (TC0), confluence was ∼80% (Figure 3A). On tissue culture‐treated plates (TC8), uninduced cultures underwent cell elongation and produced early myotubes after 8 days (TC8i), whereas induced cultures demonstrated significantly more pronounced myotube formation. Fever elongated cells were observed on gelatin, peanut, and quinoa‐coated plates (see G8i, P8i, and Q8i). However, elongated cells were observed regardless of the coating. As with C2C12 myoblasts, iBSCs are required to undergo differentiation into muscle fibers. Differentiation in vitro was conducted under the same conditions as for C2C12 myoblasts. At 80% confluence, the culture medium was replaced with a differentiation medium containing 2% (v/v) FBS, rather than the original 20% (v/v), in order to induce cell differentiation. Twelve days after induction, highly elongated iBSCs indicative of myotube formation were observed on all plates (Figure 3A). Vitronectin‐coated surfaces supported myotube formation, with the number of myotubes being consistent. Highly elongated iBSCs were also observed on the gelatin, peanut, and quinoa protein extract‐coated surfaces at 12 days of induction, indicating myotube formation under all coatings (Figure 3).

These microscopic observations were further corroborated by diagnostic reverse transcription polymerase chain reaction (RT‐PCR) analysis of myogenic marker expression (Figure 3B,C). In C2C12 cells, MyoD transcripts were barely detected at TC0 or day 8, with no apparent differences among the coating conditions. The MyoG transcript was not detected at TC0 but was visible on day 8 under all coating conditions, with comparable band intensities. Similar to MyoD, MyoHC produced very faint bands on day 8 under all conditions, with no detectable band present on TC0.

Myogenic differentiation of iBSCs on the protein extract coating was also evaluated over a 12‐day period using diagnostic RT‐PCR. MyoD transcripts were detected at day 0 on vitronectin‐coated plates (VTN0) as well as for all coating conditions at day 12 (Figure 3C). MyoG transcripts were more abundant on day 12 under the different coating conditions compared to VTN0, where only a faint band was observed. MyoHC transcript was not detected on day 0 (VTN0), but strong bands were detected on different coating conditions on day 12, indicating the coatings support cell differentiation (Figure 3C). In the experiments with C2C12 and iBSCs, transcript expression of GAPDH was used as a loading control, which showed equal loading of the samples (Figure 3B,C).

Since the diagnostic RT‐PCR provided only a qualitative assessment of myogenic marker expression and did not allow reliable determination of the relative changes in transcript abundance, we subsequently performed RP‐qPCR to quantitatively assess MyoHC expression in C2C12 myoblasts and iBSCs (Figure 3D,E). For C2C12 cells, MyoHC expression was normalized to the non‐induced day 8 (TC8) control. Surprisingly, the MyoHC transcript levels were highest in the induced TC8i, compared to the TC8 (Figure 3D). Cells cultured on the different coated surfaces also showed a modest increase in MyoHC transcript expression compared to TC8 (∼1.2–1.5‐fold, Figure 3D). For iBSCs, MyoHC transcript expression was similarly normalized to the corresponding non‐induced control (VTN12). An approximate 1.5‐fold increase in MyoHC expression was observed in most of the coating conditions except for quinoa coatings, which showed only about a 1.1‐fold increase in MyoHC transcript expression (Figure 3E).

In summary, the coatings promoted myogenic differentiation, as evidenced by the expression of myogenic marker transcripts. However, under the conditions tested, the plant‐based protein coatings did not result in a significant increase in the expression of myogenic markers.

### 3.4 Plant‐Protein Coatings Promote Protein Expression of Myogenic Markers

**FIGURE 4:**
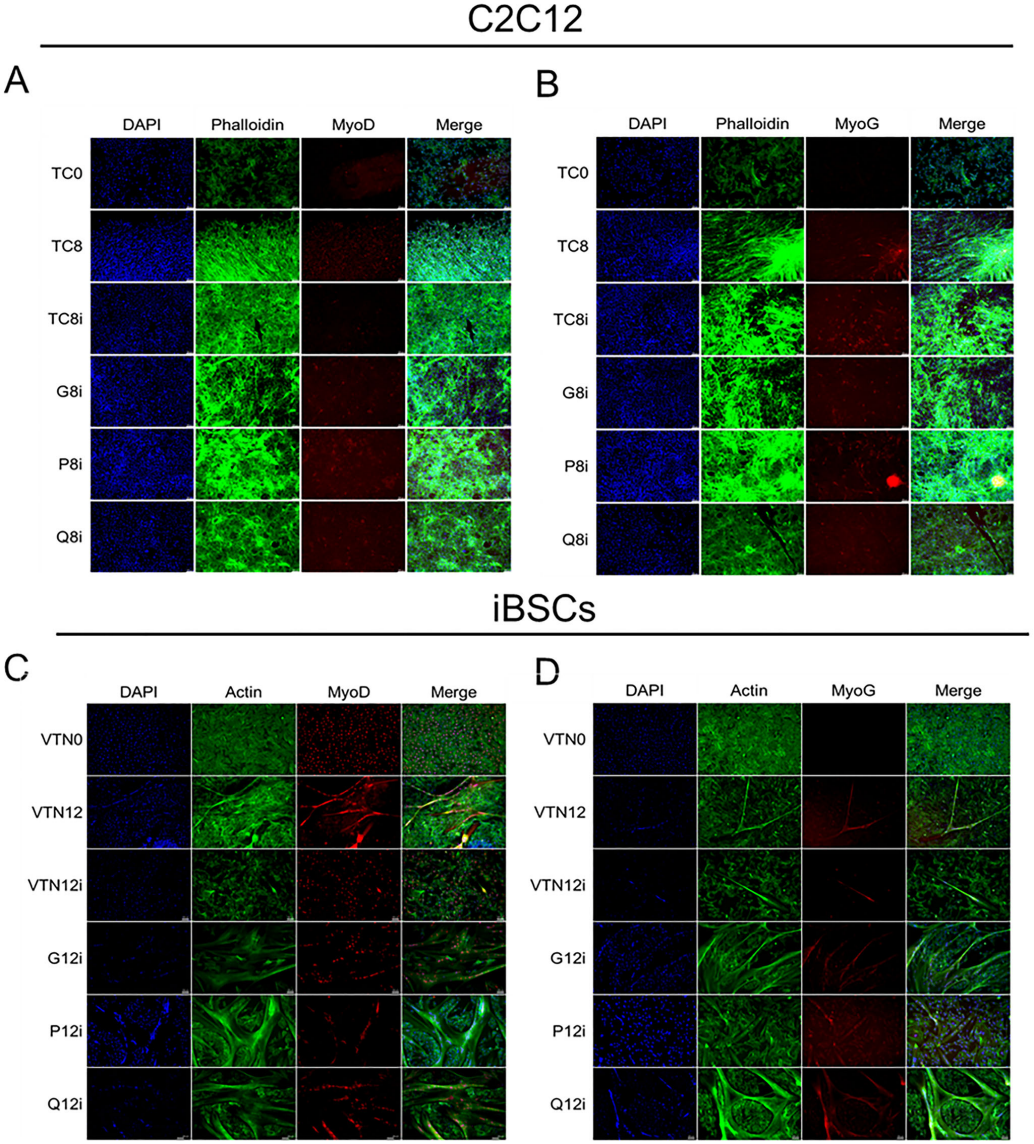
Differentiation of C2C12 myoblasts and iBSCs on peanut‐ and quinoa‐extract‐coated surfaces assessed by immunofluorescence analysis of myogenic markers. Immunofluorescence staining was utilized to label MyoD‐positive C2C12 (A) and iBSCs (C) as well as MyoG‐positive C2C12 (B) and iBSCs (D) following myogenic differentiation. The cell nuclei were counterstained with DAPI (blue), while filamentous actin (F‐actin) was stained with phalloidin. TC: Tissue culture, G: Gelatin coating, P: Peanut extract coating, Q: Quinoa extract coating, VTN: Vitronectin coating, at day 0 or day 8 after induction (i). Scale bar: 100 µm.

Finally, immunofluorescence staining was used to asses myogenic differentiation at the protein level (Figure 4). Phalloidin staining, which labels F‐actin within the cell cytoskeleton, revealed elongated cell morphologies that indicate differentiation, and DAPI staining of the cell nuclei confirmed that the cultures had reached complete confluence (Figure 4A,C). MyoD immunofluorescence signals were detected in both C2C12 cells and iBSCs under all coating conditions, as well as in the undifferentiated controls (Figure 4A,C). In contrast, MyoG immunofluorescence signals in the differentiated cells were more pronounced under the various coating conditions compared to the respective undifferentiated controls (Figure 4B,D), which is consistent with the induction of myogenic differentiation. MyoHC labeling was exclusively detected in C2C12 cells cultured on Petri dishes coated with peanut protein extract, whereas only a faint MyoHC signal was detected in the remaining samples (see Figure S2).

### 3.5 Proteomic Analysis Identifies RGD‐Containing Candidate Proteins in Peanut and Quinoa Extracts

Having demonstrated that plant seed protein extracts support the attachment, proliferation, and differentiation of mouse and bovine muscle cells, we hypothesized that cell attachment may be associated with the presence of RGD peptides in the extract, as RGD inhibition at high concentration reduced cell attachment on peanut coated‐plates (Figure S3). We next sought to identify candidate proteins that could contribute to these effects. To this end, the protein composition of the extracts was analyzed by mass spectrometry.

**FIGURE 5:**
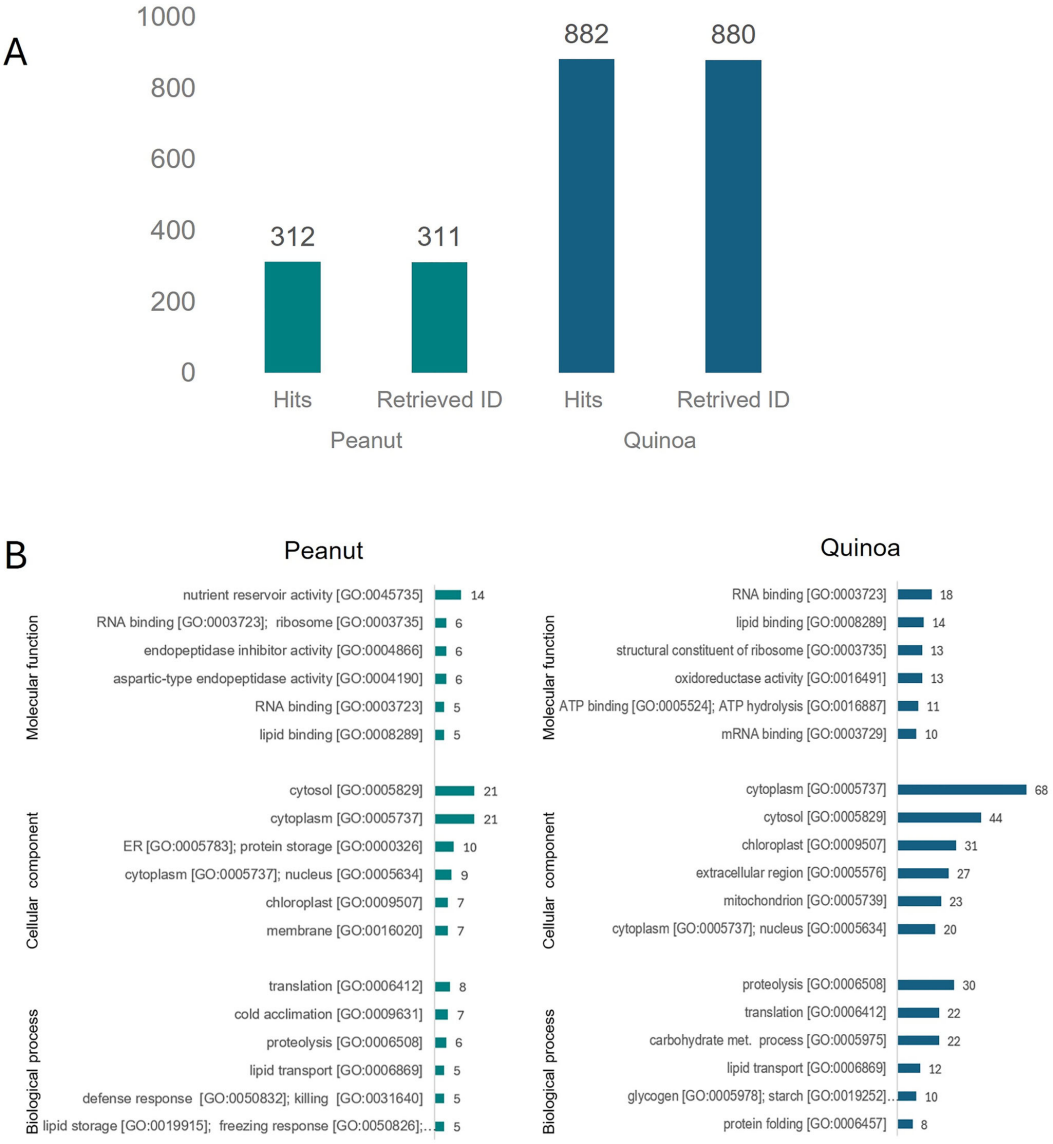
Identification and functional classification of proteins in water‐soluble peanut and quinoa extracts. (A) Total protein hits and proteins successfully identified following data retrieval and ID mapping. (B) Gene Ontology (GO) analysis of proteins identified in the extracts, categorized according to molecular function, cellular component, and biological process. For each GO category, only the six most highly represented terms are shown, and the numbers indicate the number of proteins in each GO term. For mass spectrometry, 3 independent biological samples (*N*_b_ = 3), each with 3 technical replicates (*N*_t_), were analyzed.

A total of 313 protein hits were detected in the peanut seed extract and 882 in the quinoa seed extract. After entering these hits into the UniProt Retrieve/ID Mapping Tool, 311 peanut proteins and 880 quinoa proteins were successfully identified (Figure 5A, Table S2). The identified proteins were then subjected to a Gene Ontology (GO) analysis to characterize their associated molecular functions, cellular components, and biological processes (Figure 5B, Table S2).

In the peanut seed extract, the most common molecular functions for the detected proteins included nutrient storage activity (14 proteins), RNA binding/ribosome‐associated functions (6), and endopeptidase inhibitor activity (6). With regard to cellular components, the proteins were predominantly associated with the cytosol (21), the cytoplasm (21), and the storage protein compartments of the endoplasmic reticulum (10). The most common biological processes were translation (8), cold acclimation (7), and proteolysis (6).

In the quinoa seed extract, the predominant molecular functions were RNA binding (18), lipid binding (14), and ribosome‐associated functions (13). The most common cellular components were the cytoplasm (68), the cytosol (44), the chloroplast (31), and the extracellular region (27). The most important biological processes included proteolysis (30), translation (22), carbohydrate metabolism (22), and lipid transport (12).

Overall, the GO analysis revealed distinct functional profiles between the two seed extracts. Peanut proteins showed a strong representation of nutrient storage and protease‐inhibitory functions, consistent with the accumulation of storage and protective proteins in seeds. In contrast, the quinoa proteome displayed a broader functional distribution, with substantial representation of proteins involved in protein turnover, carbohydrate metabolism, lipid binding and transport, and translation.

The 15 most abundant proteins from each extract were determined (Table 2) and were analyzed to identify known cell adhesion‐associated motifs, with a particular focus on the Arg‐Gly‐Asp (RGD) motif. Among the abundant peanut proteins, a bifunctional inhibitor containing an RGD motif was identified (A0A444Y5I1). In addition, two proteins contained an LDV motif, reported to be involved in cell adhesion by recognizing integrin α4β1 (Makarem and Humphries 1991). The abundant proteins identified in the peanut extract also included several well‐characterized peanut allergens, notably Ara h 6, Ara h 2, and Ara h 1 (Table 2).

**TABLE 2:** Most abundant proteins identified by mass spectrometry and their cell adhesive motifs. Proteins selected for cell‐free expression are indicated in bold.

Peanut extract		
Unit Prot ID	Protein description	Cell adhesion motif
A5Z1R0	Ara h 6	1 x LDV
A0A445BYI5	Ara h 2 allergens	1 x LDV
N1NG13	Allergen Ara h I	
A0A445EAK4	Uncharacterized protein	
**A0A444Y5I1**	**Bifunctional inhibitor/plant lipid transfer protein**	**1x RGD**
E5FHY8	Late embryogenesis abundant protein group 3 protein	
A0A444YT96	Oleosin	
E5FHY9	Late embryogenesis abundant protein group 4 protein	
Q2VMU0	Serine protease inhibitor	
B3IXL2	Allergen Ara h I	
B6CEX8	Non‐specific lipid‐transfer protein	
A0A445CPR0	Cupin type‐1 domain‐containing protein	
A0A445EM48	Desiccation‐related protein PCC13‐62	
A0A444XC25	Late embryogenesis abundant protein group 3 protein	
A0A445D2R2	Dehydrin	

Quinoa extract		
Unit Prot ID	Protein description	Cell adhesion motif
A0A803L851	Chitin‐binding type‐1 domain‐containing protein	
A0A803LU90	Uncharacterized protein	
A0A803MK85	Uncharacterized protein	
A0A803LYI9	Bifunctional inhibitor/plant lipid transfer protein	
A0A803MCQ9	Chitin‐binding type‐1 domain‐containing protein	
**A0A803M7P7**	**Cupin type‐1 domain‐containing protein**	**2 x RGD, 1x LDV**
A0A803L3E3	Non‐specific lipid‐transfer protein	
A0A803M7Q1	Cupin type‐1 domain‐containing protein	
Q06AW1	11S seed storage globulin B	
A0A803L3E7	Non‐specific lipid‐transfer protein	
A0A803MHX8	Non‐specific lipid‐transfer protein	
A0A803MFJ7	Cupin type‐1 domain‐containing protein	1x LRE
A0A803KW13	Chitin‐binding type‐1 domain‐containing protein	
A0A803MCQ8	Chitin‐binding type‐1 domain‐containing protein	
A0A803M3Q4	Oleosin	

Among the 15 most abundant quinoa proteins, a cupin‐type‐1 protein containing two RGD motifs and an LDV motif was identified (A0A803M7P7). Another Cupin type‐1 domain‐containing protein (A0A803MFJ7) contained an LRE motif, a cell adhesive motif in proteins of the neuromuscular junction (Johnson and Moore 2013).

**FIGURE 6:**
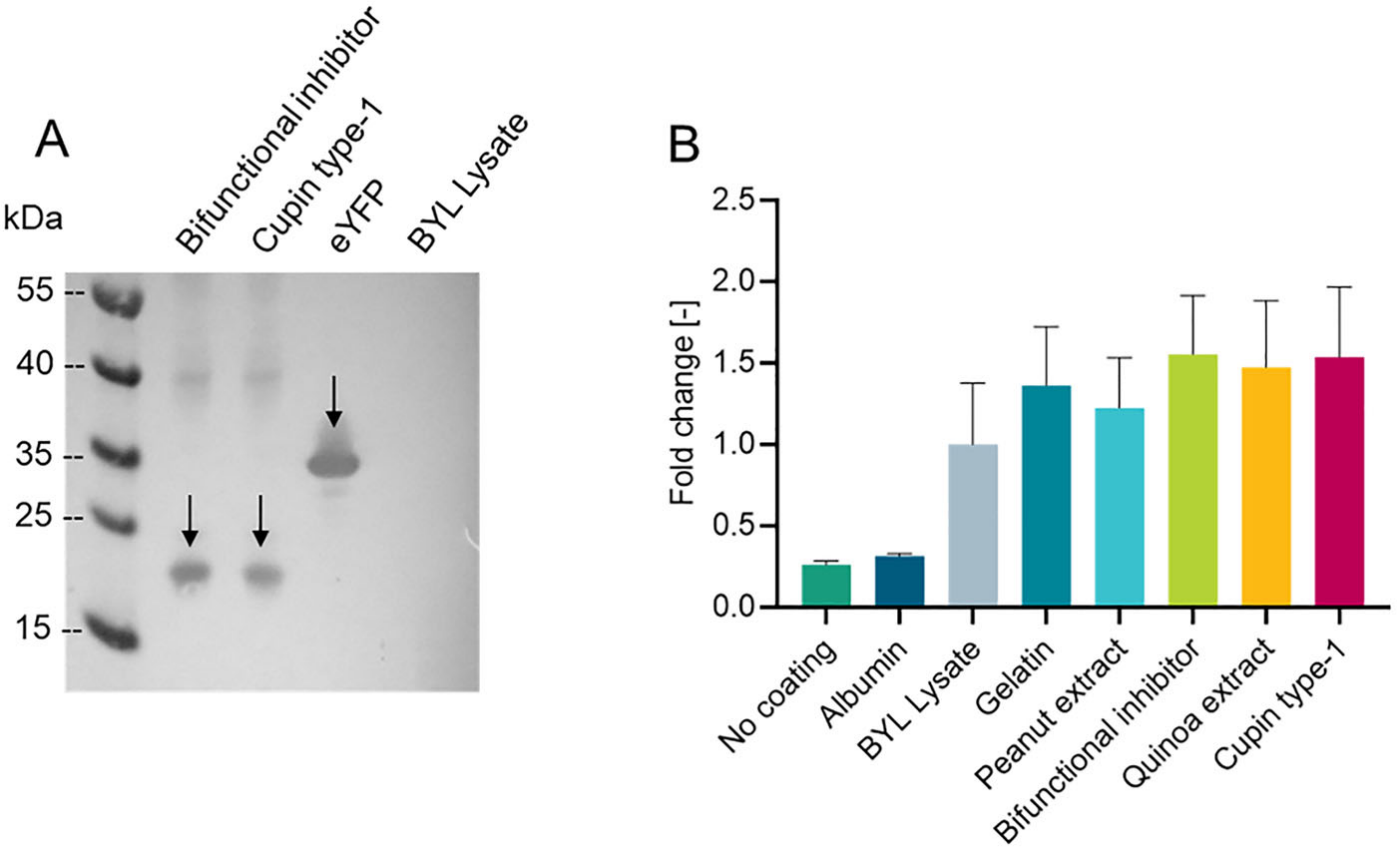
Cell‐free production of RGD‐containing candidate proteins and their characterization for cell attachment and proliferation. (A) Western blot detection of cell‐free produced plant proteins in BYL using Strep‐Tactin‐based detection. (B) Cell attachment and proliferation of iBSCs on Petri dishes coated with cell‐free produced plant proteins in BYL, as determined by the CCK‐8 assay after five days of culture. The results were normalized to the BYL control and presented as mean ± standard deviation. *N*_b_ = 3.

The two RGD‐containing motif proteins from …, x and y, were therefore selected for recombinant protein production and subsequent functional characterization. The coding sequences of the selected RGD‐containing candidates (Figure S4, S5) were cloned into the pLenEx vector via Gibson assembly and expressed using the cell‐free BYL expression system derived from tobacco cells. Due to the size of the full‐length Cupin type 1 protein, a defined fragment containing the RGD motifs of interest was selected for expression. Based on the predicted amino acid sequence, the molecular masses were approximately 19 kDa for the bifunctional inhibitor and 21 kDa for Cupin‐type I (Figure S4). Cell‐free expression of the candidate proteins was then verified by western blot. Detection using Strep‐Tactin revealed distinct bands that is consistent with the predicted molecular masses of the cell‐free‐produced proteins tagged with the strep‐tag. An eYFP construct, added as an expression control, produced a band at approximately 35 kDa (Figure 6A), confirming the functionality of the cell‐free expression system.

To investigate whether the cell‐free produced RGD‐containing proteins could reproduce the effects observed with protein extracts from whole seeds, proliferation assays were conducted under standardized coating conditions. The surfaces were coated with BYL, gelatin, peanut protein extract, BYL containing the produced bifunctional inhibitor, quinoa protein extract, BYL containing the produced cupin type 1, or albumin, with an uncoated surface serving as an additional control. The cell‐free‐produced bifunctional inhibitor and Cupin type 1 proteins were applied without purification and therefore remained in the BYL during surface coating.

Subsequent metabolic analysis of the proliferation readout revealed an increase in cell attachment of the recombinant protein‐containing lysates, but this was not significant when compared with the BYL control lacking any produced protein (Figure 6B). Therefore, the cellular effects cannot yet be attributed exclusively to the individual RGD‐containing proteins; rather, they may be due to the combined effect of several proteins or adhesion‐promoting motifs within the cell‐free extract.

## 4 Discussion

We have demonstrated that surface coatings derived from plant protein extracts, specifically from peanut and quinoa seeds, can support the adhesion and proliferation of C2C12 myoblasts and iBSCs to a degree commensurate with gelatin (for C2C12 cells) and vitronectin (for iBSCs). These findings suggest that plant proteins are suitable as animal‐free alternatives to traditional adhesion‐promoting substrates, particularly where biocompatibility, sustainability, and the avoidance of animal‐derived components are required. Several plant proteins have already gained attention as alternatives to animal adhesion substrates. For example, zein from corn (Limaye et al. 2024) and proteins derived from pumpkin seeds (Kong and Huang 2023) have been reported to support cell adhesion and growth. Limaye et al. (2024) demonstrated the suitability of zein‐based scaffolds for supporting cell growth. Cell proliferation on the scaffolds was evaluated by quantifying cell numbers using the PicoGreen assay, while cell viability was assessed using the luminescence‐based CellTiter‐Glo Luminescent Cell Viability Assay. These findings indicate that zein can provide a plant‐derived scaffold capable of supporting cell attachment and growth over a 14‐day culture period. Kong and Huang (2023) extracted proteins from a range of different plant sources, including soybeans, mung beans, red lentils, rapeseeds, peas, chickpeas, pumpkin seeds, oats, broad beans, and chia seeds, using pH precipitation. The resulting plant protein extracts were used to coat alginate microcarriers and subsequently cross‐linked with genipin. C2C12 myoblasts onto the coated microcarriers were then determined after 1 and 3 days using the CCK‐8 assay (Kong and Huang 2023). Their results demonstrated that plant proteins can be used to functionalize otherwise non‐adhesive biomaterial surfaces and thereby promote myoblast attachment and growth.

Although previous studies have demonstrated the potential of plant‐derived proteins, including zein and pumpkin seed proteins, to support mammalian cell adhesion and growth, the present study extends this concept by investigating peanut and quinoa protein extracts as alternatives to animal‐derived extracellular matrix protein for cultured meat production.

In the present study uncoated polystyrene culture surfaces and albumin coatings served as negative controls to assess non‐specific cell adhesion. C2C12 myoblasts and iBSCs exhibited low adhesion to both surfaces, with limited proliferation and predominantly round, non‐physiological morphologies. Albumin was selected as a negative control because it lacks classical integrin‐binding motifs such as RGD (Sivaraman and Latour 2010). The observed poor adhesion on albumin‐coated surfaces supports the assumption that integrin‐mediated cell adhesion occurs primarily via RGD‐containing ligands (Uhlig et al. 2014). In contrast, gelatin also contains a high amount of RGD‐peptides (Li et al. 2018) and was found to facilitate robust adhesion and proliferation for C2C12 cells, in agreement with previous studies (Wang et al. 2012). For iBSCs, vitronectin, a glycoprotein in blood plasma (Wang et al. 2006), was used as a positive control because it is known to mediate strong adhesion (Tragoonlugkana et al. 2024). Other well‐known peptides, such as Tyr‐Ile‐Gly‐Ser‐Arg (YIGSR), Ile‐Lys‐Val‐Ala‐Val (IKVAV), Pro‐His‐Arg‐Ser‐Asn (PHRSN), Asp‐Gly‐Glu‐Ala (DGEA), and Pro‐Arg‐Ala‐Arg‐Ile (PRARI), can also promote cell adhesion (Huettner et al. 2018).

Peanut and quinoa extracts were comparable to gelatin in promoting the adhesion and proliferation of C2C12 cells under our experimental conditions. Quantitative proliferation assays revealed an increase in cell count of up to 40–50% on gelatin and peanut coatings compared to uncoated controls, with no evidence of toxicity within the concentration range we tested. Conversely, a decline in cell count was observed on quinoa‐coated surfaces at the highest tested concentration, suggesting a possible concentration‐dependent inhibitory effect. One potential explanation may be the presence of high levels of residual saponins in the quinoa protein extract. Saponins are naturally occurring secondary metabolites in quinoa, predominantly located in the outer layers of the seed, and have been reported to exhibit membranolytic and cytotoxic properties depending on their concentration (Savarino et al. 2022; Xue et al. 2020). Their amphiphilic nature can facilitate interactions with membrane components and, under certain conditions, compromise membrane integrity, which may adversely affect cell viability and proliferation. Therefore, an increased amount of residual saponins associated with the higher quinoa coating concentration could potentially have contributed to the observed reduction in cell number. However, as the saponin content of the protein extracts was not quantified in the present study, this mechanism remains speculative. In addition, concentration‐dependent changes in coating properties, such as protein aggregation, surface coverage, protein conformation, or accessibility of cell‐interacting sites, may also have contributed to the observed response.

Although none of the plant‐based coatings were comparable to vitronectin in iBSC adhesion tests, quinoa coating achieved ∼75% of the cell count on the vitronectin surface and thus outperformed the peanut coating. This observation should be interpreted with caution because our experimental design did not fully control for potential differences in protein adsorption, surface coverage, and spatial protein distribution. Future studies should determine coating homogeneity and the amount of adsorbed protein, as well as systematically characterize surface properties in order to disentangle these influences.

The characterization of protein in the extracts provides a contextual framework for the interpretation of the biological findings. The results from SDS‐PAGE profiles revealed that peanut extracts predominantly contained proteins of 10–25 kDa, with a prominent band at ∼15 kDa, whereas quinoa extracts primarily contained proteins of 10–15 kDa, accompanied by weaker bands up to ∼70 kDa. The banding patterns were stable during centrifugation and filter sterilization, suggesting there was no selective removal of specific fractions. Noteworthy, batch to batch variation in the protein composition of the peanut and quinoa seeds obtained from the local retail stores was not investigated. This represents a potential limitation of the study, as previous reports have shown that the protein profiles of peanuts can be influenced by several factors such as cultivar, growing and storage conditions, processing history, and seed maturity (Wang et al. 2024).

In addition to adhesion and proliferation, we investigated myogenic differentiation. Morphological analysis revealed that following the induction of differentiation with horse serum (C2C12) or the removal of FBS (iBSCs), the formation of myotubes was possible on surfaces coated with peanut or quinoa proteins. However, compared to standard tissue culture plates, differentiation was less pronounced, as demonstrated with *MyoG* and *MyoHC* transcript expression. Immunofluorescence analysis confirmed the presence of these early differentiation markers but showed no coating‐dependent enhancement. In C2C12 cells, the decline in *MyoG* expression concomitant with a modest rise in *MyoHC* levels indicated that differentiation was not fully advanced within the 8‐day observation period. This is consistent with reports that MyoG peaks before late markers such as MyoHC (Schmidt et al. 2019). In iBSCs, a 12‐day differentiation period led to a non‐significant increase in the expression of late myogenic markers. Early markers were detectable at the protein level, but late markers were not. It is therefore evident that extended differentiation periods or optimized induction protocols will be necessary to reliably assess the differentiation‐related effects of plant‐derived coatings.

To initially assess whether RGD motifs could be involved in cell attachment, peanut‐coated plates were incubated with cells pretreated with RGD peptides at different concentrations to block integrin‐binding sites. The treated cells were subsequently cultured on the peanut‐coated plates. Cell numbers decreased at higher RGD peptide concentrations, suggesting that RGD‐mediated interactions may contribute to cell attachment to the extracts. To further investigate whether the extracts contained proteins harboring RGD motifs that could mediate cell attachment, quinoa and peanut protein extracts were prepared and subjected to mass spectrometry analysis. The analysis identified numerous proteins in both the peanut (311) and quinoa extracts (880) after screening.

In the peanut extract, proteins such as Ara h 2 and Ara h 6, were among the most abundant proteins in the extracts. These proteins are among the most clinically relevant peanut allergens and are characterized by their high stability against heat, changes in pH, and proteolytic degradation (Lehmann et al. 2006).

Although no proteins specifically classified as allergens were detected in the quinoa extracts, quinoa seeds have been reported to contain proteins with allergenic potential (Ballegaard et al. 2023). Thus, the absence of identified allergens in the present mass spectrometry analysis does not necessarily exclude the presence of allergenic proteins in the quinoa extracts. The potential presence of allergenic proteins in both peanut and quinoa extracts represents an important consideration when evaluating their suitability as food‐grade coating materials for cultured meat.

This concern is particularly relevant for peanut‐derived coatings, as peanut is a well‐established allergenic food source and the peanut extract contained substantial amounts of Ara h 2 and Ara h 6. These proteins are major peanut allergens and have been associated with severe allergic reactions (Lehmann et al. 2006). Consequently, the incorporation of peanut‐derived extracts into edible coatings could raise allergen‐related safety concerns and require appropriate allergen risk assessment, management, and labeling. Although quinoa is generally considered to have a lower allergenic potential than peanut, allergic reactions to quinoa and IgE‐reactive quinoa proteins have been reported (Ballegaard et al. 2023). Therefore, the possible presence of allergenic proteins should also be taken into account when assessing quinoa‐derived extracts as food‐grade biomaterials.

Importantly, the identification of proteins by mass spectrometry alone does not establish whether they retain allergenic activity in the resulting coating or final cultured‐meat product. The actual allergenic risk may depend on several factors, including the identity and abundance of the allergenic proteins, processing conditions, structural integrity, digestibility, and the amount of coating material retained in the final edible product. Therefore, further studies are required to determine the immunoreactivity of the extracts, the resulting coatings, and ultimately the final cultured meat product, for example, using allergen‐specific immunoassays. In addition, further processing, fractionation, or purification of the extracts could be explored to determine whether the components responsible for promoting cell adhesion can be retained while reducing or eliminating potentially allergenic proteins. Such investigations will be important for establishing the safety and practical suitability of these plant‐derived extracts for food‐grade applications.

Further analysis of the mass spectrometry data was performed to determine whether the peanut and quinoa extracts contained proteins harboring RGD motifs that could potentially contribute to the observed cell attachment. This analysis identified an RGD‐containing bifunctional inhibitor in the peanut extract (UniProt: A0A444Y5I1) and a cupin type‐1 domain‐containing protein in the quinoa extract (UniProt: A0A803M7Q1). Both proteins were detected at relatively high abundance, making them potential candidates for contributing to the cell‐adhesive properties of the respective extracts.

To obtain initial insights into whether these RGD‐containing proteins could promote cell attachment, protein regions containing the RGD motif were produced using the tobacco BYL cell‐free protein synthesis system described by (Buntru et al. 2022; Schillberg et al. 2019), and the resulting protein‐containing lysates were evaluated as coating materials. Bifunctional inhibitors are plant defense proteins that can exhibit both serine protease‐ and α‐amylase‐inhibitory activities (Basso et al. 2025; Bonturi et al. 2022). In contrast, cupin type‐1 domain‐containing proteins belong to the structurally diverse cupin superfamily, which includes major seed storage proteins such as 7S and 11S globulins that serve as nutrient reserves during seed germination and early seedling development (Dunwell et al. 2004; Zhu et al. 2023).

Proliferation assays showed that C2C12 cells were able to attach and proliferate on coatings prepared from lysates containing the recombinant RGD‐containing protein regions. The observed effects were within the range obtained with the corresponding seed protein extracts. However, no statistically significant increase in cell numbers was observed compared with the BYL lysate control. Importantly, the BYL itself supported cell attachment and proliferation, suggesting that endogenous components of the BYL may provide a background that promotes cell adhesion and growth. This background effect may have reduced the ability to distinguish a specific contribution of the recombinant RGD‐containing proteins.

Another possible explanation for the lack of a statistically significant effect is that the recombinant proteins were tested directly within the complex BYL reaction mix rather than as purified proteins. Consequently, the concentration and surface accessibility of the RGD‐containing proteins in the coating may have been insufficient to generate a measurable additional effect over that of the lysate itself. Purification of the recombinant proteins, followed by coating at defined concentrations, would therefore be required to more directly assess their cell‐adhesive properties. Moreover, the presence of an RGD sequence alone does not establish that the motif is functionally accessible or capable of mediating integrin‐dependent adhesion when embedded within a particular protein structure.

Finally, the cell‐adhesive activity observed for the peanut and quinoa extracts is unlikely to be attributable exclusively to the identified RGD‐containing proteins. The extracts represent complex mixtures containing numerous proteins and other biomolecules that may independently or synergistically influence surface properties, cell attachment, and proliferation. Other adhesive peptide motifs or RGD‐independent interactions may therefore contribute to the effects observed with the crude extracts. Further experiments using purified RGD‐containing proteins, appropriate RGD‐negative or motif‐mutated controls, and integrin‐blocking approaches would be necessary to establish whether the identified RGD motifs directly mediate C2C12 cell attachment. Thus, the present findings identify these proteins as plausible candidates but do not yet demonstrate a causal relationship between their RGD motifs and the cell‐adhesive activity of the peanut and quinoa extracts.

## 5 Conclusion

This study demonstrates the potential of plant‐derived protein extracts as food‐grade coating materials to support cell attachment, proliferation and differentiation for cultured meat applications. Peanut and quinoa extracts promoted the attachment, proliferation, and differentiation of C2C12 and iBSC cells, indicating that these extracts may provide a promising alternative to conventional animal‐derived or purified extracellular matrix components for cell culture applications.

Proteomic analysis revealed a diverse protein composition in both extracts and identified abundantly expressed proteins containing RGD motifs, including an RGD‐containing bifunctional inhibitor in peanut and a cupin type‐1 domain‐containing protein in quinoa. Protein regions containing these motifs were subsequently produced using a tobacco BYL cell‐free system and evaluated for their ability to support C2C12 cells. Although cell attachment and proliferation were observed with the corresponding protein‐containing lysates, the effects were not significantly different from those obtained with the BYL control lacking any cell‐free produced protein product. Therefore, the present results do not establish a direct causal relationship between the identified RGD‐containing proteins and the cell‐adhesive properties of the seed extracts. Other proteins, adhesive motifs, or components within the complex extracts may contribute individually or synergistically to the observed effects.

At the same time, the presence or potential presence of allergenic proteins represents an important consideration for the translation of these materials to food applications, particularly for peanut‐derived extracts containing major allergens such as Ara h 2 and Ara h 6. Future studies should therefore focus on identifying the specific components and mechanisms responsible for cell adhesion, including experiments with purified RGD‐containing proteins and appropriate functional controls, while simultaneously assessing the immunoreactivity and food safety of the resulting coatings and final products.

Overall, these findings provide a basis for the development of plant‐derived, food‐compatible coatings for cultured meat production and highlight both the opportunities and challenges associated with using protein extracts. Further optimization and characterization may enable the development of defined plant‐derived coating formulations that combine effective cell adhesion with scalability, food compatibility, and an acceptable safety profile.

## Nomenclature

ECM: Extracellular matrix
FBS: Fetal bovine serum
iBSC: Immortalized bovine satellite cell
MyoD: Myoblast determination protein 1
MyoG: Myogenin
MyoHC: Myosin heavy chain
P/S: Penicillin/Streptomycin
RT: Room temperature
SDS‐PAGE: Sodium Dodecyl Sulfate Polyacrylamide Gel Electrophoresis
% (v/v): Volume by volume percentage
% (w/v): Weight per volume

## Author Contributions

**Dominik Mundt**: conceptualization, methodology, data curation, software, investigation, validation, formal analysis, writing – original draft, writing – review and editing, visualization. **Kuo–Hui Chiu**: methodology, supervision, investigation, writing – review and editing, visualization. **Stefan Schillberg**: conceptualization, supervision, resources, project administration, funding acquisition, writing – review and editing. **Che Julius Ngwa**: conceptualization, methodology, project administration, supervision, resources, funding acquisition, writing – review and editing.

## Conflicts of Interest

The authors declare no conflicts of interests.

## Supporting information




**Supplementary Materials**: jfds71509‐sup‐0001‐SuppMat.docx

**Supplementary Materials**: jfds71509‐sup‐0002‐TableS2.xlsx
