# Supplementary material for: Quinoa and Peanut Extracts as Alternatives to Animal‐Derived Extracellular Matrix Proteins in Cultured Meat Production
Source: J Food Sci. 2026 Sep 27;91(10):e71509. doi: 10.1111/1750-3841.71509 (PMC13616198; doi:10.1111/1750-3841.71509)
Supplement: Supplementary file 1 [file JFDS-91-0-s002.docx]

Journal of Food Science: Original Research Manuscript Template

Quinoa and peanut extracts as alternatives to animal-derived extracellular matrix proteins in cultured meat production

Dominik Mundt^1^, Kuo-Hui Chiu^1^, Stefan Schillberg^1,2^ and Che Julius Ngwa^1*^

Supplemental Information

Table S1: Primers used for diagnostic and quantitative RT-qPCR analysis.

| Primer Name | 5′→3′ (Forward) | 3′→5′ (Reverse) |
| --- | --- | --- |
| C2C12 MyoD | GGCTACGACACCGCCTACTAC | GGTCTGGGTTCCCTGTTCTG |
| C2C12 MyoG | CCTGGAAGAAAAGGGACTGG | CGCTCAATGTACTGGATGGC |
| C2C12 MyoHC | CGCAAGAATGTTCTCAGGCT | GCCAGGTTGACATTGGATTG |
| C2C12 GAPDH | GTATGATGACATCAAGAAGGTG | TCCTTGGAGGCCATGTAGGCCAT |
| iBSCs MyoD | CAACGCCATCCGCTATATC | GCCGCTGTAGTCCATCAT |
| iBSCs MyoG | AGAAGGTGAATGAAGCCTTCGA | GCAGGCGCTCTATGTACTGGAT |
| iBSCs MyoHC | GCCCACTTCTCCCTCATTCACT | ACCCTTCTTCTTGCCACCTTTC |
| iBSCs β-actin | ATGATATTGCTGCGCTCGTGG | TACGAGTCCTTCTGGCCCAT |

Fig. S1: Representative image of C2C12 myoblasts after attachment assay on plant protein-coated Petri dishes over 3 days and determination of optimal extract protein concentration. A: Representative image of C2C12 myoblasts after cultivation on plant protein, gelatin-coated Petri dishes over 5 days. B: Determination of optimal coating concentration of gelatin, peanut extract and quinoa extract. Cells were cultivated over 3 days. Cell number was determined by CCK-8 assay. Results are normalized to non-coated petri dishes (0 µg/cm^2^) and are given as +/- standard deviation. N_t_ = 3.

Fig.S2: Differentiation of C2C12 and iBSCs on peanut- and quinoa-extract coated surfaces assessed by protein expression analysis of myogenic marker. Immunofluorescence staining was utilized to label MyoHC-positive C2C12 (A) and iBSCs (B) DAPI staining, which results in a blue hue, was utilized to label cell nuclei. Phalloidin, a fluorescent stain that labels filamentous actin, was utilized in this study. TC: Tissue culture, G: Gelatin coating, P: Peanut extract coating, Q: Quinoa extract coating, VTN: Vitronectin coating, At Day 0 or days 8 after induction. Scale bar: 100 µm.

Fig. S3: RGD inhibition assay using coated plates. (A) RGD inhibition assay using peanut extract coated plates. (B) RGD inhibition assay using gelatin coated plates as positive control. Plates were coated with the respective proteins, peanut extract (0.5% w/v) gelatin (1% w/v). Albumin (1% w/v) was used as negative control. Afterwards, C2C12 were incubated with different concentrations of RGD to block integrin binding sites. Following treatment, the cells were washed with PBS cell pellets were resuspended in fresh culture medium and seeded onto the pre-coated plates. Cells were incubated for 3 h at 37 °C to allow adhesion. After incubation, the wells were gently washed with PBS to remove non-adherent cells. The number of adherent cells was subsequently quantified by measuring the absorbance using the CCK-8 assay.

Fig. S4: Schematic presentation of the amino acid and DNA sequences of the identified proteins containing cell-binding peptides. P: Cloned sequence of Bifunctional inhibitor with 1 RGD sequence identified in peanut extract used for cell-free expression. Q: Identified Cupin type-1 with 2 RGD sequences and 1 LDV sequence identified in quinoa extract (blue) and cloned fragment with 2 RGD sequences using BYL cell-free expression system (orange).

Figure S5: Genblock for cell-free expressed RGD containing plant proteins. Gene blocks of the bifunctional inhibitor identified in the peanut extract and the Cupin type-1 gene block identified in the quinoa extract. The gene blocks were cloned into the pLenEx-239 vector using Gibson assembly followed by cell-free production of the target proteins in the cytosolic fraction of the BYL system. Restriction sites for ‘NcoI’ and ‘KpnI’ flanking the gene fragments were used for cloning.

Formula S1: Calculation of optimal plasmid concentration for cell-free protein expression using the BYL expression system

$$V \left[ \mu L \right]=Plasmid length \left[ bp \right]\times618\frac{g}{mol \times bp} \times Reaction volume \left[ \mu L \right]\times\frac{Final concentration [nM]}{Plasmid stock [ng/\mu L]} \times{10}^{-6}$$
